# Nowcasting tourist nights spent using innovative human mobility data

**DOI:** 10.1371/journal.pone.0287063

**Published:** 2023-10-13

**Authors:** Umberto Minora, Stefano Maria Iacus, Filipe Batista e Silva, Francesco Sermi, Spyridon Spyratos

**Affiliations:** 1 European Commission, Joint Research Centre, Ispra, Italy; 2 Institute for Quantitative Social Sciences, Harvard University, Cambridge, MA, United States of America; Babes-Bolyai University, Cluj-Napoca, ROMANIA

## Abstract

The publication of tourism statistics often does not keep up with the highly dynamic tourism demand trends, especially critical during crises. Alternative data sources such as digital traces and web searches represent an important source to potentially fill this gap, since they are generally timely, and available at detailed spatial scale. In this study we explore the potential of human mobility data from the Google Community Mobility Reports to nowcast the number of monthly nights spent at sub-national scale across 11 European countries in 2020, 2021, and the first half of 2022. Using a machine learning implementation, we found that this novel data source is able to predict the tourism demand with high accuracy, and we compare its potential in the tourism domain to web search and mobile phone data. This result paves the way for a more frequent and timely production of tourism statistics by researchers and statistical entities, and their usage to support tourism monitoring and management, although privacy and surveillance concerns still hinder an actual data innovation transition.

## Introduction

Tourism is an important economic activity in many countries and regions globally, providing economic and development opportunities for residents and opportunities for recreation, personal and cultural enrichment, business and networking for tourists. In the European Union, prior to the COVID-19 pandemic, the travel and tourism sector contributed to more than 11% of the employment when considering direct, indirect, induced and catalytic effects [1]. However, the contribution of tourism to employment can vary from as little as 5% in some Eastern European countries to nearly 20% in Croatia or Malta [1], and its heterogeneous geographical distribution is even more evident at regional and local levels [2].

The varying importance of tourism by country and region relates to their characteristics and attractiveness as tourist destinations, pulling tourism demand. The attractiveness of destinations depends on more or less stable factors, ranging from climate [3], presence of socio-economic, cultural and natural assets and events offering opportunities for tourism [4], but also transport connectivity. Marketing and social media influencing strategies are increasingly shaping tourists’ preferences and, thus, tourism demand too [5]. Tourism is also affected by seasonality, which is often constrained by school and business calendars [6, 7], but with a strong regional variation too, depending on the type of tourism offer and the climate conditions of destinations [2, 8]. The knowledge of factors shaping tourism demand is important for tourism management by the industry and for public policy [9].

However, tourism demand can be rapidly affected by exogenous shocks of different nature, such as socioeconomic downturns, wars, terrorism, epidemics and other natural or man-made catastrophes [10–12]. COVID-19 and the Russian-Ukrainian war are recent and eloquent examples. Travel and tourism were among the most hit economic activities following the outbreak of the COVID-19 in early 2020 [13] and the mobility and other socio-economic restrictions put in place by governments worldwide in an attempt to contain the spread of the SARS-CoV2 virus [14]. Beyond the COVID-19 pandemic mobility restrictions, surveys have shown a shift in consumer preferences and decision-making [15, 16], including tourists preferences away from mass and urban destinations, and towards more rural or nature-related travel, active tourism, and trips aimed at health recovery [17–19]. In addition, the drop in tourism demand is not evenly distributed across Europe in 2020 [20]. Instead, it depended on geographical and other region-specific factors, even after controlling for the COVID-19 stringency measures.

The publication of official tourism statistics does not keep up with the speed of tourism dynamics. Often, such statistics are published months if not years after the fact, and as aggregated (areal) measures, under-mining the efficiency of the decision-making processes [21]. This is especially the case for statistics at higher spatial granularity [22]. For example, at the time of writing (August 2022), tourism demand statistics for European countries compiled by Eurostat such as the monthly number of nights spent or arrivals are available until April 2022 on a per country basis, and yet with some missing values. At sub-national level the gap is greater, with the latest data points for the year 2020 published in mid-2022, and without monthly breakdown, according to data from Eurostat (last accessed: 2022–07-25). Such gaps between the date of the events and the availability of data limits the capacity of decision makers in both industry and the public sector to respond timely and adequately to rapidly changing trends and their potential consequences.

In this paper, we address this gap by proposing a new machine learning implementation to nowcast tourism demand at the sub-national level. Literature on nowcasting and forecasting is relatively abundant and not new [23] (see Section Literature review). Nevertheless, to the best of our knowledge, the implementation herein introduces two novel elements in relation to the state-of-the-art: 1) the use of an alternative predictor data source, concretely the Google Community Mobility Reports, and 2) its testing under the specific context of the COVID-19 shock, including the initial shock (2020) and subsequent recovery years (2021, 2022).

The Google Community Mobility Reports (GCMR) product was launched in 2020 amidst the early stages of the COVID-19 pandemic, to help health authorities monitor mobility trends in their countries and regions. The dataset covers the period from February 2020 to mid-October 2022, and historical data are publicly available [24]. GCMR are based on users of Google mobile product ecosystem, tracking individuals movements throughout the day, to obtain insights about mobility on an aggregate, anonymized format, broken down by sub-national regions and by different categories of places such as retail and recreation, groceries and pharmacies, parks, transit stations, workplaces, and residential areas.

Google mobility data have been used in several studies [25–27], but, to the best of our knowledge, not yet for predicting tourism demand. Our research question is whether the monthly trends in human movements that can be detected within regions using the GCMR can be a reliable (co-)predictor for monthly tourism demand, as an alternative to the popularized web search data. Our hypothesis is that the number of tourists visiting regions are reflected in observed mobility (*i.e*. people moving within a region). This is coherent with the notion of tourism being a form of mobility itself [28], and with the expectation that the presence of additional people in a given region adds to demand for inner-regional mobility.

The study is of empirical nature, consisting on the development of a machine learning implementation using Google mobility data and other control variables to predict observed nights spent. To test the model, we compare our estimates with known values of nights-spent per region and look at the explanatory power of the predictor variables uses (*i.e.* variable importance). We carry this study in a multi-country environment and during the years between 2020 and 2022 in which tourism demand was affected by COVID-19 in an unprecedented way, making this both a novel and challenging exercise.

In the next section we carry out a literature review, focused mainly on documented approaches and data to analyse and predict tourism demand with innovative data. The section Materials and methods describes the data sources and methods used in this study, followed by a section documenting the obtained results. In the Discussion of the estimation error we analyze the model performance, we present our results in relation to other known approaches from the literature, and we reflect upon any privacy concerns about the mobility data and beyond. In the last section, Conclusions, we wrap up the key takeaways and implications of our study for the production of more frequent and timely tourism demand estimates.

### Literature review

According to the meta-analysis by [29], the first studies on forecasting and nowcasting tourism demand originate in the early 1980’s. Tourism demand forecasting is usually performed using non-causal time series, econometric, and artificial intelligence-based approaches [30]. However, very limited academic research has been conducted into tourism forecasting using big data due to the difficulties in capturing, collecting, handling, and modeling this type of data, which is normally characterized by its privacy and potential commercial value [31]. Nevertheless, they provide several benefits over traditional data and methodologies such as increasing the sample base on which conventional research tends to be based by several orders of magnitude [32], and providing real-time information and nowcasting [33].

For methods involving explanatory or predictor variables, web search data has become a recurrent choice [30, 34–36], even for forecasting at high spatial resolution such as municipalities [22], and sometimes in combination with economic (*e.g.* prices) data [37]. Aggregated Google Trend data for Hong Kong’s tourism demand forecasting suggested that Google Trends’ data about a destination may be useful in predicting visits to that destination [38]. Visitor numbers for a popular tourist destination in China were predicted using web search query volume, with a significant decrease in forecasting errors when search engine data were used [39]. However, there is contrasting evidence on the benefit of including web search data for improving tourism related forecasts. For instance, the contribution of lagged Google Trend variables in a standard ARIMA model and in a time series regression model with seasonal dummies and autoregressive components did not seem to add significant contribution in nowcasting the monthly number of foreign arrivals in Italy [34]. Moreover, in forecasting tourist volumes with search trend data, one needs to collect tourism-related keywords, obtain their search trend data, select appropriate data series to construct an aggregated index, and construct econometric models. The major challenges are keyword selection and search data aggregation [40].

In a parallel strand of literature, researchers have been experimenting with geolocalised mobile phone data to obtain more granular (spatially and temporally) measurements of tourism flows to and within a tourism destination [41, 42], opening up possibilities also for closer to real-time assessments. During the COVID-19 emergency, a unique Business-to-Government initiative was established to stream the European Commission with anonymized human mobility data derived from mobile network operators in Europe, to understand the spread of the disease, the effectiveness of the containment measures and their socio-economic impacts [43], a task which does not come without several data harmonisation and governance challenges [44]. However, in the field of tourism the use of mobile phone data is still very limited in terms of countries and domain, and only a few wide-ranging examples are available [42]. Mobile positioning data form mobile phones of foreign visitors were used for measuring visitor flows to destinations in Estonia from 2011 to 2013 and they are used by the Estonian Tourist Board [41]. BPS-Statistics Indonesia has used mobile positioning data for official statistics since October 2016 [45]. The literature review of the recent contributions to the use of mobile phone data in quantifying the volume of tourist flows and a brief case study of the Metropolitan City of Florence in [46] showed the main weaknesses of mobile phone data, which include costs, privacy restrictions, statistical issues of representativeness, among others.

In some recent studies [47, 48] the possibility of using Google Location History (GLH) data to characterise fine-scale human mobility was investigated, and the results suggest that they could provide unmatched individualised human movement information and also address some key gaps in data that are currently available. In the next section, we describe how we used this data together with tourism indicators to nowcast nights spent across 11 European countries in 2020 to 2022 at sub-national scale.

## Materials and methods

### Research design for tourism nowcast

Our aim is to nowcast monthly tourism nights spent across a set of selected European countries at sub-national (NUTS3) scale between January 2020 and July 2022 using tourism indicators, population data, and human mobility data from the GCMR.

To capture the relationships between tourism nights spent (*N*, the dependent or response variable), and the selected predictors, we apply a machine learning approach using Random Forests [49]. We try to fit the following model for each country separately:

$$\begin{array}{c} \dot{N_{t}}=\text{RandomForest}\left(G_{t},P,V,T\right) \end{array}$$ (1)

where *t* is a month in the available time window, *V* and *T* are tourism indicators (vulnerability and typology classes respectively), *P* is the population data, and *G* is a mobility indicator deriving from the GCMR.

The motivation for using Random Forest is that it is a flexible and robust model designed to capture high non-linearities (if any) between a dependent variable and the covariates. One important feature is its use of out-of-bag (OOB) samples [50], which allows to measure the prediction errors using a set of observations which is not involved in the training process of the model, and as such can be used to validate its accuracy. OOB also allows to measure the predictive power of each variable. We make use of the Random Forest implementation in the R package ranger [51].

To understand the predictive power of the selected covariate, we investigate the variable importance scores provided by the Random Forest algorithm. The values of importance are obtained from the OOB sample and represent the reduction in sum of squared errors whenever a variable is chosen to split a node across every tree of the forest. Since we fit a different model for each available country, each importance value is divided by the sum of all values found in all variables, to make them comparable across countries.

To select the optimal parameters for the model, we perform hyperparameter tuning with the grid search method, evaluating the OOB errors, which correspond to the Mean Squared Error (MSE) in the case of regression model using the R package ranger. The only dynamic hyperparameter we consider in the tuning phase is the number of variables to possibly split at in each node (mtry), for which we set a bottom limit equivalent to the square root of the number of variables, while the upper limit considers all available variables. We also increase the default number of trees (parameter num.trees in the ranger function) from 500 to 2500 as we expect better results using a higher value [52], without the risk of overfitting [49]. Since all but the mobility indicator are static variables, the assumption that we are testing is that if the model is able to capture the temporal and seasonal variability in the number of nights spent, we should conclude that it is the mobility factor which explains this variation. In other words, after we control for the structural components of tourism and the population, the rest of the variability is controlled by the mobility indicator.

Table 1 shows the sub-national areas that we are able to cover combining all the selected data sources.

**Table 1:** Available NUTS3 areas by country.

	N	%
Croatia	21	100
Czech Republic	14	100
France	96	95
Hungary	20	100
Italy	101	91
Lithuania	6	60
Luxembourg	1	100
Romania	42	100
Slovakia	8	100
Spain	43	72
Sweden	21	100

For the population data we use the “population on 1 January by age group, sex and NUTS 3 region” dataset at NUTS3 spatial resolution as of 2020 from Eurostat available at https://appsso.eurostat.ec.europa.eu/nui/show.do?dataset=demo_r_pjangrp3&lang=en (last accessed: 15/02/2022). The remaining dataset are described in the following sections, which also explain the transformation some of them required to be used in the final model.

### Tourism data

Tourism indicators at sub-national (NUTS3) resolution were provided by [8]. They include:

- Monthly total nights spent in 2018 at sub-national (NUTS3) scale (*i.e.* the response variable);
- Tourism typology: reflects the geographical context of the accommodation capacity;
- Vulnerability class, in 5 levels (the higher, the more vulnerable). This index takes into account three indicators: tourism intensity, tourism seasonality and share of foreign tourists;

The availability of nights spent at sub-national scale allows us to have a sufficient number of observations to perform a stronger meaningful statistical analysis compared to what we could do with other available statistics at country level. Unfortunately, these are only available for 2018, whereas the GCMR are available since February 2020. Therefore we developed a strategy to rescale the sub-national nights spent to the study period using recent tourism data at national scale from Eurostat (last accessed: 01/09/2022).

To rescale the nights spent we first aggregate the sub-national value to national ones in 2018 for each country *c* and month *m* (*N*_2018,*m*,*c*_) by summing the values in all sub-national areas *k* as:

$$\begin{array}{c} N_{2018,m,c}=\sum_{i=1}^{K}N_{2018,m,k} \end{array}$$ (2)

where *i* is the *i* − *th* sub-national area in country *c* and *K* the number of total areas.

Then we calculate a national rescaling factor *f* which is the ratio of the total nights spent in one month between 2020 and 2022 (*N*_*y*,*m*_), and the aggregated value in 2018 in the same month.

$$\begin{array}{c} f_{y,m}=\frac{N_{y,m}}{N_{2018,m}} \end{array}$$ (3)

where *y* is one year between 2020 and 2022.

Finally, for each country, we obtain the sub-national nights spent rescaled to 2020 to 2022 as:

$$\begin{array}{c} {\dot{N}}_{y,m,k}=N_{2018,m,k}\cdot f_{y,m} \end{array}$$ (4)

Eq 4 implies that the values of nights spent are rescaled by monthly factors based on the ratio between national totals in two different years, assuming a proportional rescaling for all NUTS3 regions. This is a simplistic assumption that can be further improved using other forms of weighting but, as the accuracy of the results will show, its impact is mild and further improvement would increase the model complexity with likely little gain in accuracy.

### Google mobility data

In order to capture human mobility, we make use of Google Community Mobility Reports at NUTS3 level, which covers the period from February 2020 to mid-October 2022 and are publicly available [24]. They derive from Google Location History data and they come in an aggregated and anonymized form, from users who had turned on the Location History setting (more on this will be discussed in Section Privacy concerns and availability of Google mobility data). They are provided with daily frequency and are grouped into different categories of places, each with similar characteristics for purposes of social distancing guidance. These place categories are:

- *Retail and recreation*: Mobility trends for places like restaurants, cafes, shopping centers, theme parks, museums, libraries, and movie theaters.
- *Grocery and pharmacy*: Mobility trends for places like grocery markets, food warehouses, farmers markets, specialty food shops, drug stores, and pharmacies.
- *Parks*: Mobility trends for places like national parks, public beaches, marinas, dog parks, plazas, and public gardens.
- *Transit stations*: Mobility trends for places like public transport hubs such as subway, bus, and train stations.
- *Workplaces*: Mobility trends for places of work.
- *Residential*: Mobility trends for places of residence.

The above indicators represent a positive or negative percentage of change in mobility compared to a baseline day, as described at https://support.google.com/covid19-mobility/answer/9824897?hl=en&ref_topic=9822927# (last accessed: 16/02/2022). All indicators have similar trends and they are highly correlated with each other, with the *Residential* category showing an opposite pattern as compared to the other categories. This means their information is somwehat redundant (see the correlation matrices in S1 Appendix). Moreover, the indicators are not always available for a given date and/or a given NUTS3 region.

To limit the gaps within the series and at the same time avoid model specification issues due to multiple correlation, we take advantage of the common scale of the indicators to derive a single synthetic mobility indicator, *G*. To do this, we first multiply each observation from the *Residential* category by -1 so that its pattern is similar to the other indicators, in the sense that it conveys the same type of information (*i.e.* when people stay home, their overall mobility in other destination decreases). Then we derive *G* as the mean of the six indicators *X*_*i*_ in a given day *d* and NUTS3 *k*:

$$\begin{array}{c} G_{k,d}=\frac{\sum X_{i,k,d}}{I} \end{array}$$ (5)

where *I* is the number of original indicators.

**Fig 1:**
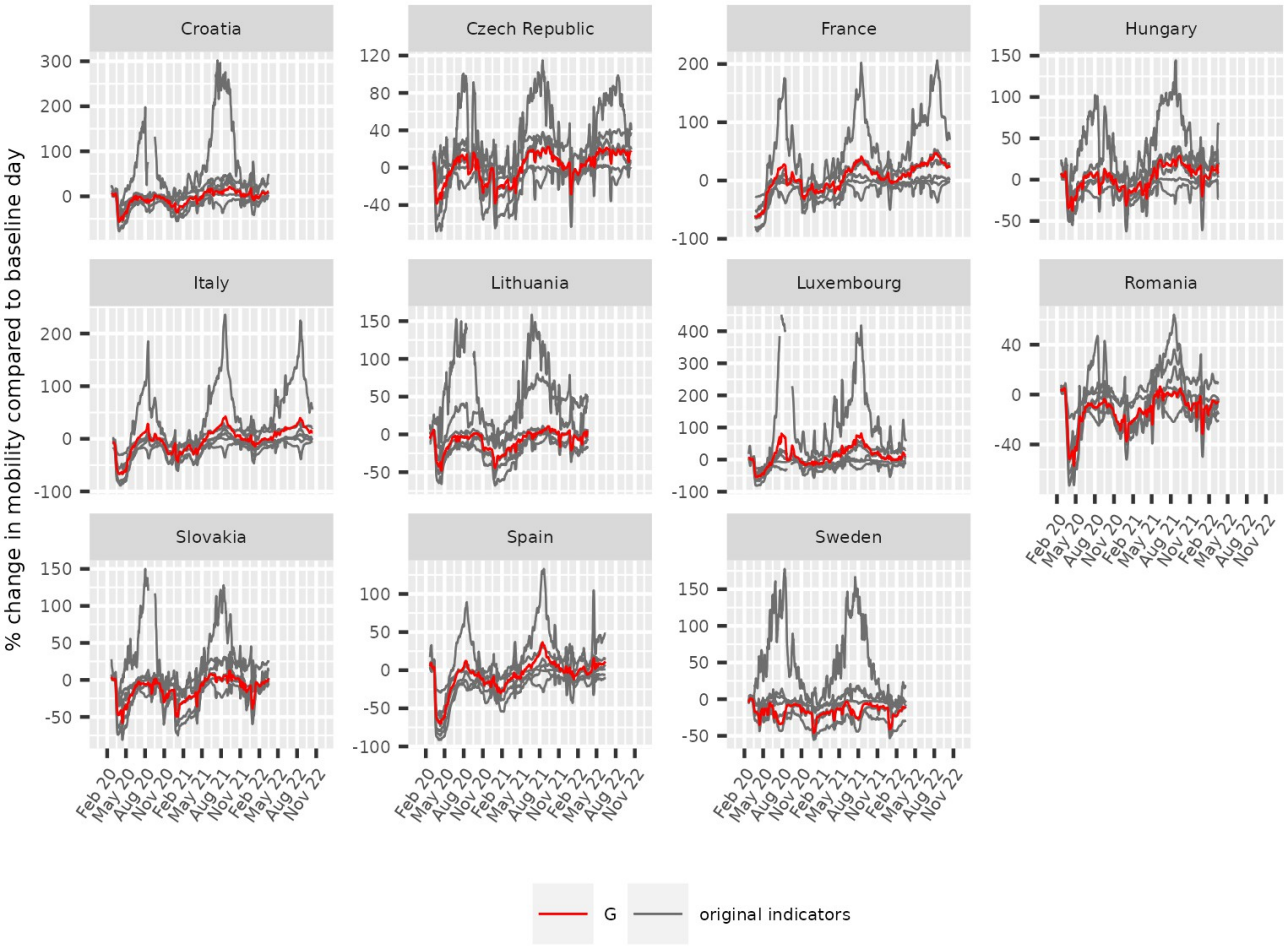
Mobility indicator time series. Percentage change in mobility compared to the baseline day in each available country. Light grey: original indicators; Red: *G*.

Fig 1 shows the resulting synthetic indicator *G* in red, and the original ones in light grey for each available country. For visualization purposes, we take the daily median of all regional indicators in each country, to which we further apply a seven days rolling average to smooth the effects of the weekends on the time series. The figure and the tables in S1 Appendix show how well the derived indicator incorporates the overall signal of the original indicators, while filling the gaps in the time series as much as possible.

As we are interested in aggregating the daily observations to match the monthly resolution of the nights spent dataset, having less gaps allows the final derived indicator to have enough observations to cover (represent) a month. Even so, gaps still exist, so to prevent bias in estimating the total mobility for a given month we further rescale *G* to the full month using a scaling factor *c* based on the number of the days in a given month and the actual observations of *G* available in the same month:

$$\begin{array}{c} c_{y,m,k}=\frac{\#\text{days}\;\text{in}\;G_{y,m,k}}{D_{y,m}}, \end{array}$$ (6)

where *D*_*y*,*m*_ is the number of days in month *m* of year *y*. Finally, we rescale the monthly mobility indicator as follows:

$$\begin{array}{c} {\dot{G}}_{y,m,k}=\frac{1}{c_{y,m,k}}\cdot \sum_{d\in D_{y,m}}G_{y,m,k,d} \end{array}$$ (7)

By taking the monthly sum of the daily mobility rather than the mean with the idea of approximating the total number of movements in a month to be compared to the total number of nights spent, we should avoid diluting seasonal information in the data.

**Fig 2:**
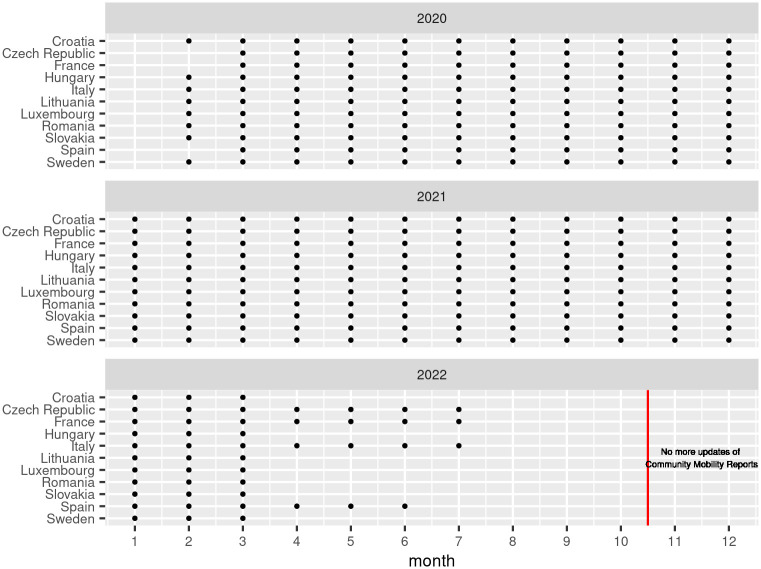
Data availability. Availability of the Google mobility indicator by month, country and year.

Fig 2 shows the overall availability of the mobility data by country and by month. The only year where we have full coverage in all countries is 2021. 2020 is also well covered, and data availability starts in February 2020, since this is when Google started sharing the mobility reports after the spread of the COVID-19 pandemic. As of the time of writing we can fully cover the first half of 2022 with the available data.

Google mobility data do not contain the official NUTS3 codes from Eurostat as present in the other variables. Therefore we map the Google administrative names to the NUTS3 codes using the COVID19 R package by [53], and the regions package as a fallback [54].

## Results

### The predictive power of human mobility

**Fig 3:**
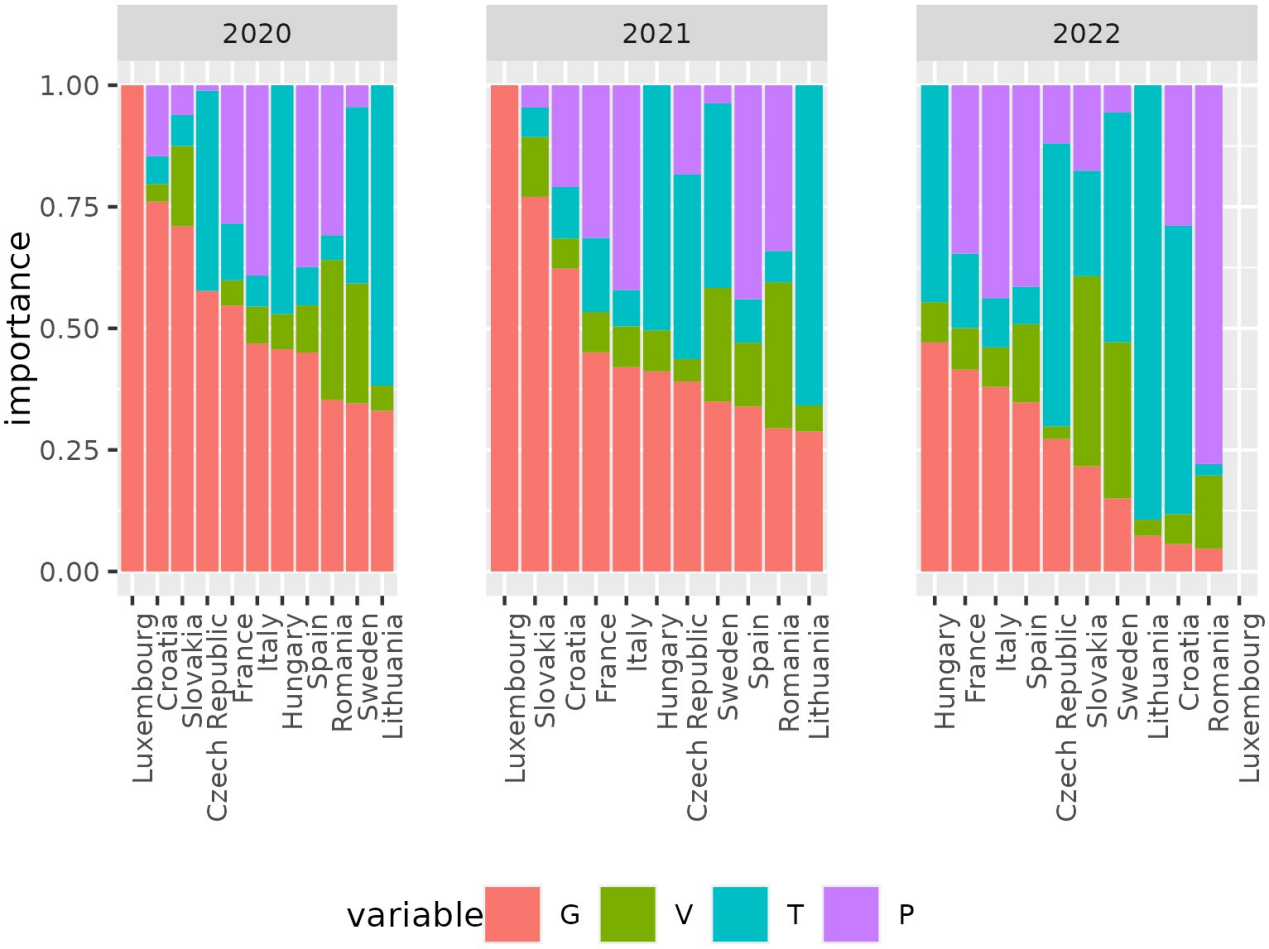
Variable importance. Variable importance plot for all countries in 2020 to 2022.

Fig 3 shows for each country and year the variable importance score, that is, a measure of the predictive power of each feature which is available in Random Forests [49, 55].

It is clear that for 2020 and 2021 *G* has a greater importance compared to the other features in predicting nights spent. In general, the results for 2022 are different than other years. This might be because we don’t cover a whole year of observations as in the other cases (see also Fig 2). Nevertheless, the relative importance of *G* is still quite high compared to the other covariates. Values for Luxembourg in 2022 are omitted as none of the predictor variables is informative for the response, or, in other words, all predictors are equally uninformative. This is because Luxembourg has no sub-national subdivisions (see Table 1), and a coverage of only three months in 2022 means a total of only three observations is available to train the model, which is too few. For 2020 and 2021 *G* is the only variable considered by the Random Forests in Luxembourg. This implies that the other static predictors (*tourism indicators and population*), which have the same value in all observations, cannot be used to model the nights spent in this country.

### Evaluation of the estimation error

We aggregate (sum) the results from sub-national to national level so we can compare them with data from Eurostat which are country level statistics, and evaluate the estimation error of the model. We calculate two types of error: a daily sub-national estimation error (*Err*_*abs*), and a relative estimation error (*Err*_*rel*).

*Err*_*abs* represents the average estimation error of the predictions per region and day in absolute units. In other words, it represents the number of nights spent per day per region that our model was not able to predict on average. It is calculated as follows:

$$\begin{array}{c} \text{Err}\_{\text{abs}}_{y,c}=\frac{N_{y,c}-{\dot{N}}_{y,c}}{D_{y,c}}\cdot {\frac{1}{K}}_{c} \end{array}$$ (8)

where *N*_*y*,*c*_ and ${\dot{N}}_{y,c}$ are respectively the nights spent observed and predicted in year *y* and country *c*, *D*_*y*,*c*_ is the number of days (*i.e.* observations) available in year *y* and country *c*, and *K*_*c*_ is the number of available NUTS3 regions in country *c* (see Table 1 for the details on NUTS3 availability).

The other type of estimation error, the relative estimation error or *Err*_*rel*, provides a vision of the relative weight of the absolute error for each country in percentage units. It is calculated as follows:

$$\begin{array}{c} \text{Err}\_{\text{rel}}_{y,c}=\frac{N_{y,c}-{\dot{N}}_{y,c}}{N_{y,c}}\% \end{array}$$ (9)

**Fig 4:**
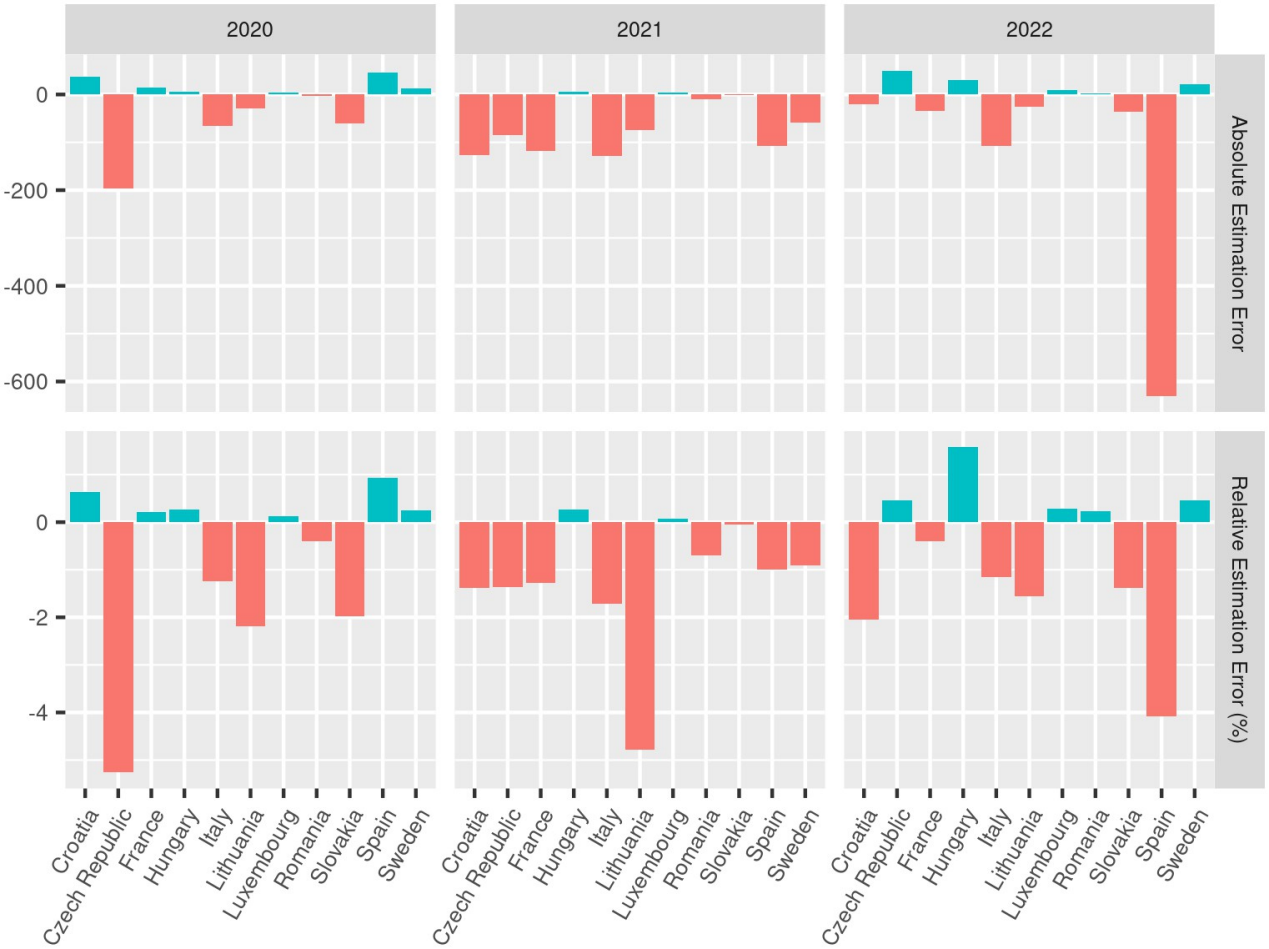
Estimation error. Absolute and relative estimation errors of nights spent for each country and years 2020 to 2022.

Fig 4 shows both the nights spent estimation errors for each country and years 2020 to 2022. Remark that values for 2022 do not refer to a whole year of observations (also see the data availability for 2022 and other years in Fig 2).

Table 2 shows the estimation errors along with the respective model error (*Mod*_*Err*). We calculate the Root Mean Squared Error (RMSE) using the OOB errors, and then we derive *Mod*_*Err* as the values of RMSE relative to the total observed nights spent:

$$\begin{array}{c} Mod\_Err=\frac{RMSE_{y,c}}{N_{y,c}}\% \end{array}$$ (10)

**Table 2:** Absolute and relative estimation errors and relative model errors for each country in year 2020, 2021, and 2022. The first column shows the available countries with ISO 3166–1 alpha-2 country codes (https://www.iso.org/iso-3166-country-codes.html. Last accessed the 2022/05/16).

	*Err*_*abs*			*Err*_*rel*%			*Mod*_*Err*%		
	2020	2021	2022	2020	2021	2022	2020	2021	2022
Czech Republic	-196.1	-84.9	+49.6	-5.3	-1.4	+0.5	+1.0	+1.0	+1.2
Spain	+45.8	-107.3	-629.7	+0.9	-1.0	-4.1	+0.3	+0.3	+0.4
France	+15.0	-118.8	-34.6	+0.2	-1.3	-0.4	+0.1	+0.1	+0.1
Croatia	+36.6	-126.0	-20.4	+0.6	-1.4	-2.0	+1.3	+1.3	+0.8
Hungary	+5.0	+6.4	+30.8	+0.3	+0.3	+1.6	+0.7	+0.7	+0.9
Italy	-66.6	-128.5	-107.3	-1.2	-1.7	-1.1	+0.2	+0.2	+0.2
Lithuania	-29.8	-74.9	-25.5	-2.2	-4.8	-1.6	+1.7	+1.6	+3.3
Luxembourg	+4.8	+3.7	+9.1	+0.1	+0.1	+0.3	+7.2	+5.0	+11.7
Romania	-3.6	-9.3	+2.0	-0.4	-0.7	+0.2	+0.4	+0.3	+0.4
Sweden	+12.7	-58.3	+22.0	+0.2	-0.9	+0.5	+0.4	+0.4	+1.3
Slovakia	-61.3	-1.1	-37.0	-2.0	-0.0	-1.4	+1.2	+1.2	+1.4

Overall, all predictions have a very low relative error (|*Err*_*rel*| < 5%), with the exception of the Czech Republic in 2020, which is still very low (*Err*_*rel* = −5.3%, see Table 2).

## Discussion

### Model performance across different countries

Despite the relative error of our model being generally relatively low (|*Err*_*rel*| < 5%), we do observe that for some countries the performance are worse than others. We argue that this might be due to the low number of NUTS3 areas in these countries, meaning that less observations are available to train the model. For Lithuania for example, which has only six areas, our model underestimates the nights spent at national level by −4.8% in 2021. Not only the number of NUTS3 areas is low in Lithuania, but our dataset also covers only 60% of the total number of NUTS3 regions in the country, which translates into an even smaller training sample. Nevertheless, the final error is still relatively small.

A peculiar case is given by Luxembourg, which has a very low relative estimation error despite having no NUTS3 subdivision. This extreme example is particularly interesting because one might argue that the accuracy of the model is not affected by the size of the training sample, since it should be worse than the Czech Republic and Lithuania on the basis of our argument. By looking at the feature importance scores for Luxembourg in Fig 3, we can see that the yearly static predictors (*P*_*k*_, *V*_*k*_, and *T*_*k*_) are not present since their value is always the same for all observations, leaving the human mobility (*G*) as the only important predictor. This probably means that *G* has a great predictive power in Luxembourg that could capture the nights spent alone quite well. Moreover, the model error in this country is much larger than the other countries, and so is its *RMSE*. It is difficult to assess the reasons behind the difference in the model accuracy for the various countries, however we do observe that our model is capable of nowcasting tourism nights spent with high accuracy overall.

We acknowledge that one major limitation of the current study is the lack of tourism nights spent data at sub-national level for the study period. Despite the overall good accuracy of the nowcasts we observe, the transformation we are doing to rescale the 2018 data to 2020–2022 has lots of implications. It assumes a constant rescaling factor for each sub-national area based on a correction factor calculated at national scale. This is particularly tricky since our analysis focuses on a period during which tourism demand was affected by COVID-19 in an unprecedented way, when regions were impacted somehow differently due to the spread of the virus and to different lockdown measures. An alternative approach to tackle the research question would have been to focus on the year 2018, but on the other side the GCMR data is only available since February 2020. We look forward to possibly check if our results still hold with data on tourism demand at sub-national scale in our study period should they become available anytime in the future.

### Google mobility data compared to other innovative data sources for tourism nowcast

Google Community Mobility Reports and, more in general, GLH data from which they derive, share some similarities in terms of potential and limitations with mobile phone data, which have already been explored extensively in many research fields including the one of tourism (see Section Literature review). On the one hand, they provide timely and quasi real-time information about human mobility, cover large populations, and can provide insights on various aspects of human behaviour that official and more traditional statistics cannot provide. On the other hand, they are not generated with the scope of providing tourism statistics, and so they include movements of residents and commuters other than visitors and tourists, they entail privacy restrictions, and statistical issues of representativeness.

One advantage of GLH data over mobile phone data like Call Detail Records is that they have been collected in an opt-out, passive fashion for Android users since location services have been fully integrated into Android in 2012 [56], and Android OS is the most popular OS in the world, with over three billion active monthly Android devices around the world as of 2021 [57]. For this reason they are more widespread than mobile phone data, whose representativeness depends on the market penetration rates of different mobile network operators in different countries. Another advantage is that they use all available means, including wire-less fidelity (Wi-Fi) positioning systems, GPS satellites, or mobile networks, to locate the device [58]. Moreover, international travels are harder to analyze using mobile phone data. These are generally studied using roaming data, which have some additional limitations like double counting due to people traveling with more than one SIM card or to the SIM card connecting to the antennas of multiple different providers in the destination country [59]. In addition, the location information of mobile roaming data might be at a relatively low level of accuracy, particularly when the Global System of Mobile communication (GSM) is poorly equipped in the tourism destinations [60].

When we compare Google mobility data with web search data, another innovative data which has been extensively explored in the field of tourism (see Section Literature review), one obvious distinction is that the volume of web search that does not directly link to tourism as opposed to GCMR data which represents actual flows of people, including tourists. Despite many works have shown that the inclusion of web search data such as Google Trend data in a nowcasting exercise can improve the accuracy of the outcomes, some others did not see any added value compared to traditional time series models (*ARIMA*) without the inclusion of such data. Moreover, the selection and collection of tourism-related keywords, and creating an aggregated indicator to be used in the final models represent key challenges in using web search data are [40].

### Privacy concerns and availability of Google mobility data

The GCMR were made available during the COVID-19 pandemic to provide insights into what changed in movement trends in response to policies aimed at combating the emergency [24]. The publication of this data was a response to the public health officials wanted to make more critical decisions to combat the COVID-19.

Location tracks data is passively collected by Android smartphones, that spans large temporal scales with high spatial granularity [47]. When enabled within Android smartphones, “*Location History*” passively and continuously collects location data using technologies that include GPS, Wi-Fi and cellular positioning. Insights in the GCMR were created with aggregated, anonymized sets of data from users who had turned on the Location History setting, which is off by default. The anonymization process includes differential privacy, a mathematical method that transforms the original data into synthetic data by adding different types of noise in a way that the results of certain statistical analysis remain valid and such that the re-identification of the original data records is virtually impossible [61–63].

Despite all these privacy safeguards, access to GLH is still very limited to the researchers, and most existing data bases like the one used in [64] are not currently available for any research goal other than for COVID-19 related studies. Moreover, according to a study on environmental health research using Google mobility data, breaches to individual privacy is still a concern for many individuals, particularly given that GLH data are different from GPS data because they are collected over long time periods where individuals may not have been aware that location data was being collected, within a device they carry on a daily basis for other purposes (*i.e.*, texting, phone calls, app usage) [65]. Therefore, privacy and surveillance issues are two key factors preventing the availability of Google mobility data in the research domain. As a matter of fact, GCMR are no longer being updated as of mid-October 2022, although all historical data remain publicly available.

## Conclusions

Tourism is an important economic activity in many countries and regions, providing economic and development opportunities for residents and opportunities for recreation, personal and cultural enrichment, business and networking for tourists. However, the publication of official tourism statistics does not keep up with the speed of tourism dynamics. Often, such statistics are published months if not years after the fact. This is especially the case for statistics at higher spatial granularity [22]. This can limit the capacity of decision makers in both industry and the public sector to respond timely and adequately to ongoing trends. In this context, innovative data such as digital traces and web searches represent an important source to potentially fill this gap, since one common advantage they share over official statistics is that they are generally timely, and can be available at detailed spatial scale.

In this work, we explore the potential of human mobility data from Google to nowcast tourism demand. This is the first research to use this type of data in the tourism domain to the best of the authors’ knowledge. Comparing this data source to other innovative sources already employed in the tourism field (*e.g.* mobile phone and web search data), GLH data has the clear benefit of being extremely widespread, while directly link to tourism, since tourism is a form of mobility.

We demonstrate that human mobility data from Google coupled with other static tourism indicators can be used to nowcast tourism demand with high accuracy. Using these data and a Machine Learning approach, we model the tourism nights spent across 11 EU Member States in 2020, 2021, and the first half of 2022 at sub-national scale. We then spatially aggregate our estimates and validate them with available official statistics at national level. Overall, our model has a very high accuracy, with errors lower than 5% a day by region on average over all countries.

One major limitation of the mobility dataset used in this study is about data availability: Google does not provide updated mobility reports since mid-October 2022 as of the time of writing. One possible reason is that the use and share of this type of data entails privacy and surveillance issues, similar to mobile phone data. We believe the present work can be an initial step forward to prove the potential and relevance of this type of human mobility data in the field of tourism nowcasting, and towards a more frequent, and spatially detailed production of tourism statistics through innovative mobility data by researchers or statistical entities, and their usage to support tourism monitoring and management more timely and thus more effectively, while waiting for official statistics to be released.

The proposed data-driven approach should provide the policymakers and managers in the field of tourism timely insights which are not possible to derive from official statistics for better decision-making and greater operational efficiencies. The key to fully unleash the potential of this and other non-traditional data sources is to create the right conditions to enable a data innovation transition, that is to say, a transition from a phase of exploratory use of innovative data to a phase of systematic use of innovative data in official statistics and policymaking. These conditions can include a regulatory framework, the development of operational models and secure technical systems, and investments aimed at fostering the collaboration between all the involved stakeholders.

## Supporting information

S1 Appendix Correlation of Google mobility indicators. Following are the correlation matrices for the original mobility indicators and the composite one (*G*) in each country. *V*1, *V*2, …, *V*6 represent the Google mobility indicators.
(ZIP)
