# Supplementary material for: Nowcasting tourist nights spent using innovative human mobility data
Source: PLoS One. 2023 Oct 13;18(10):e0287063. doi: 10.1371/journal.pone.0287063 (PMC10575538; doi:10.1371/journal.pone.0287063)
Supplement: S1 Appendix — Following are the correlation matrices for the original mobility indicators and the composite one (G) in each country. V1, V2, …, V6 represent the Google mobility indicators. (ZIP) [file pone.0287063.s001.zip › supplement.pdf]

S1 Appendix

Correlation of Google mobility indicators. Following are the correlation matrices for the original mobility indicators and the composite one (G) in each country. $V1, V2, \ldots, V6$ represent the Google mobility indicators.

Table 1. Czech Republic.

	V1	V2	V3	V4	V5	V6	G
V1	1.00	0.66	0.45	0.63	0.53	0.69	0.84
V2	0.66	1.00	0.27	0.45	0.48	0.48	0.68
V3	0.45	0.27	1.00	0.44	0.22	0.48	0.75
V4	0.63	0.45	0.44	1.00	0.52	0.62	0.80
V5	0.53	0.48	0.22	0.52	1.00	0.79	0.64
V6	0.69	0.48	0.48	0.62	0.79	1.00	0.80
G	0.84	0.68	0.75	0.80	0.64	0.80	1.00

Table 2. Spain.

	V1	V2	V3	V4	V5	V6	G
V1	1.00	0.75	0.75	0.82	0.57	0.81	0.94
V2	0.75	1.00	0.53	0.67	0.64	0.68	0.82
V3	0.75	0.53	1.00	0.62	0.25	0.54	0.85
V4	0.82	0.67	0.62	1.00	0.60	0.77	0.87
V5	0.57	0.64	0.25	0.60	1.00	0.80	0.64
V6	0.81	0.68	0.54	0.77	0.80	1.00	0.83
G	0.94	0.82	0.85	0.87	0.64	0.83	1.00

Table 3. France.

	V1	V2	V3	V4	V5	V6	G
V1	1.00	0.87	0.73	0.74	0.57	0.73	0.92
V2	0.87	1.00	0.62	0.65	0.55	0.62	0.83
V3	0.73	0.62	1.00	0.61	0.20	0.40	0.90
V4	0.74	0.65	0.61	1.00	0.53	0.66	0.83
V5	0.57	0.55	0.20	0.53	1.00	0.83	0.53
V6	0.73	0.62	0.40	0.66	0.83	1.00	0.68
G	0.92	0.83	0.90	0.83	0.53	0.68	1.00

Table 4. Croatia.

	V1	V2	V3	V4	V5	V6	G
V1	1.00	0.85	0.79	0.83	0.67	0.73	0.92
V2	0.85	1.00	0.70	0.72	0.59	0.62	0.84
V3	0.79	0.70	1.00	0.79	0.36	0.47	0.94
V4	0.83	0.72	0.79	1.00	0.59	0.63	0.92
V5	0.67	0.59	0.36	0.59	1.00	0.84	0.60
V6	0.73	0.62	0.47	0.63	0.84	1.00	0.67
G	0.92	0.84	0.94	0.92	0.60	0.67	1.00

Table 5. Italy.

	V1	V2	V3	V4	V5	V6	G
V1	1.00	0.84	0.76	0.80	0.62	0.82	0.93
V2	0.84	1.00	0.56	0.66	0.57	0.62	0.78
V3	0.76	0.56	1.00	0.73	0.32	0.57	0.92
V4	0.80	0.66	0.73	1.00	0.59	0.73	0.88
V5	0.62	0.57	0.32	0.59	1.00	0.80	0.59
V6	0.82	0.62	0.57	0.73	0.80	1.00	0.78
G	0.93	0.78	0.92	0.88	0.59	0.78	1.00

Table 6. Lithuania.

	V1	V2	V3	V4	V5	V6	G
V1	1.00	0.44	0.48	0.56	0.58	0.67	0.76
V2	0.44	1.00	0.32	0.45	0.40	0.40	0.68
V3	0.48	0.32	1.00	0.52	0.16	0.42	0.80
V4	0.56	0.45	0.52	1.00	0.39	0.48	0.80
V5	0.58	0.40	0.16	0.39	1.00	0.81	0.56
V6	0.67	0.40	0.42	0.48	0.81	1.00	0.70
G	0.76	0.68	0.80	0.80	0.56	0.70	1.00

Table 7. Luxembourg.

	V1	V2	V3	V4	V5	V6	G
V1	1.00	0.86	0.68	0.78	0.35	0.68	0.88
V2	0.86	1.00	0.54	0.63	0.37	0.60	0.77
V3	0.68	0.54	1.00	0.58	0.01	0.34	0.92
V4	0.78	0.63	0.58	1.00	0.60	0.80	0.80
V5	0.35	0.37	0.01	0.60	1.00	0.85	0.32
V6	0.68	0.60	0.34	0.80	0.85	1.00	0.63
G	0.88	0.77	0.92	0.80	0.32	0.63	1.00

Table 8. Hungary.

	V1	V2	V3	V4	V5	V6	G
V1	1.00	0.80	0.64	0.88	0.56	0.68	0.93
V2	0.80	1.00	0.33	0.67	0.51	0.49	0.73
V3	0.64	0.33	1.00	0.64	0.25	0.45	0.82
V4	0.88	0.67	0.64	1.00	0.69	0.77	0.92
V5	0.56	0.51	0.25	0.69	1.00	0.83	0.65
V6	0.68	0.49	0.45	0.77	0.83	1.00	0.75
G	0.93	0.73	0.82	0.92	0.65	0.75	1.00

Table 9. Romania.

	V1	V2	V3	V4	V5	V6	G
V1	1.00	0.83	0.54	0.53	0.42	0.73	0.85
V2	0.83	1.00	0.42	0.46	0.44	0.66	0.77
V3	0.54	0.42	1.00	0.31	0.21	0.48	0.79
V4	0.53	0.46	0.31	1.00	0.33	0.50	0.70
V5	0.42	0.44	0.21	0.33	1.00	0.67	0.54
V6	0.73	0.66	0.48	0.50	0.67	1.00	0.78
G	0.85	0.77	0.79	0.70	0.54	0.78	1.00

Table 10. Slovakia.

	V1	V2	V3	V4	V5	V6	G
V1	1.00	0.76	0.52	0.82	0.62	0.77	0.92
V2	0.76	1.00	0.23	0.58	0.53	0.52	0.71
V3	0.52	0.23	1.00	0.44	0.17	0.45	0.75
V4	0.82	0.58	0.44	1.00	0.58	0.70	0.84
V5	0.62	0.53	0.17	0.58	1.00	0.82	0.64
V6	0.77	0.52	0.45	0.70	0.82	1.00	0.80
G	0.92	0.71	0.75	0.84	0.64	0.80	1.00

Table 11. Sweden.

	V1	V2	V3	V4	V5	V6	G
V1	1.00	0.78	0.33	0.61	0.26	0.45	0.67
V2	0.78	1.00	0.27	0.47	0.18	0.27	0.57
V3	0.33	0.27	1.00	0.31	-0.01	0.29	0.85
V4	0.61	0.47	0.31	1.00	0.52	0.70	0.67
V5	0.26	0.18	-0.01	0.52	1.00	0.80	0.39
V6	0.45	0.27	0.29	0.70	0.80	1.00	0.64
G	0.67	0.57	0.85	0.67	0.39	0.64	1.00
